# Human Occipital and Parietal GABA Selectively Influence Visual Perception of Orientation and Size

**DOI:** 10.1523/JNEUROSCI.3945-16.2017

**Published:** 2017-09-13

**Authors:** Chen Song, Kristian Sandberg, Lau Møller Andersen, Jakob Udby Blicher, Geraint Rees

**Affiliations:** ^1^Institute of Cognitive Neuroscience, University College London, London WC1N 3AR, United Kingdom,; ^2^Wellcome Trust Centre for Neuroimaging, University College London, London WC1N 3BG, United Kingdom,; ^3^Department of Psychiatry, University of Wisconsin-Madison, Madison, Wisconsin 53719,; ^4^Center of Functionally Integrative Neuroscience, Aarhus University, 8000 Aarhus C, Denmark,; ^5^Hammel Neurorehabilitation and Research Centre, Aarhus University Hospital, 8450 Hammel, Denmark, and; ^6^NatMEG, Department of Clinical Neuroscience, Karolinska Institutet, 171 77 Stockholm, Sweden

**Keywords:** contextual modulation, GABA, individual differences, lateral connection, visual illusion

## Abstract

GABA is the primary inhibitory neurotransmitter in human brain. The level of GABA varies substantially across individuals, and this variability is associated with interindividual differences in visual perception. However, it remains unclear whether the association between GABA level and visual perception reflects a general influence of visual inhibition or whether the GABA levels of different cortical regions selectively influence perception of different visual features. To address this, we studied how the GABA levels of parietal and occipital cortices related to interindividual differences in size, orientation, and brightness perception. We used visual contextual illusion as a perceptual assay since the illusion dissociates perceptual content from stimulus content and the magnitude of the illusion reflects the effect of visual inhibition. Across individuals, we observed selective correlations between the level of GABA and the magnitude of contextual illusion. Specifically, parietal GABA level correlated with size illusion magnitude but not with orientation or brightness illusion magnitude; in contrast, occipital GABA level correlated with orientation illusion magnitude but not with size or brightness illusion magnitude. Our findings reveal a region- and feature-dependent influence of GABA level on human visual perception. Parietal and occipital cortices contain, respectively, topographic maps of size and orientation preference in which neural responses to stimulus sizes and stimulus orientations are modulated by intraregional lateral connections. We propose that these lateral connections may underlie the selective influence of GABA on visual perception.

**SIGNIFICANCE STATEMENT** GABA, the primary inhibitory neurotransmitter in human visual system, varies substantially across individuals. This interindividual variability in GABA level is linked to interindividual differences in many aspects of visual perception. However, the widespread influence of GABA raises the question of whether interindividual variability in GABA reflects an overall variability in visual inhibition and has a general influence on visual perception or whether the GABA levels of different cortical regions have selective influence on perception of different visual features. Here we report a region- and feature-dependent influence of GABA level on human visual perception. Our findings suggest that GABA level of a cortical region selectively influences perception of visual features that are topographically mapped in this region through intraregional lateral connections.

## Introduction

The inhibitory neurotransmitter GABA plays a central role in visual processing ranging from neural selectivity and neural response gain control to synaptic plasticity and network oscillation (Priebe and Ferster, 2008; Katzner et al., 2011; Lehmann et al., 2012). The level of GABA, measured using magnetic resonance spectroscopy (MRS), varies substantially across human individuals, and this variability may contribute to interindividual differences in visual processing and visual perception. Indeed, a higher GABA level is associated with higher visual discrimination ability, lower susceptibility to distraction, stronger surround suppression, and stronger interocular suppression (Edden et al., 2009; Yoon et al., 2010; van Loon et al., 2013; Sandberg et al., 2014, 2016; Lunghi et al., 2015). Moreover, in neurological disorders such as attention-deficit/hyperactivity disorder and schizophrenia, both an abnormal level of GABA and an abnormal performance in perceptual tasks are observed (Moult, 2009; Yoon et al., 2010; Edden et al., 2012).

The widespread influence of GABA raises the question of whether interindividual variability in GABA reflects an overall variability in visual inhibition and has a general influence on visual perception or whether the GABA levels of different cortical regions have selective influence on perception of different visual features. One hypothesis is that the GABA level of each cortical region is uniquely determined in each individual, possibly by a combination of genetic and environmental factors (Marenco et al., 2010; Taniguchi et al., 2011; Bachtiar et al., 2015; Lunghi et al., 2015). As such, the GABA levels of different cortical regions may exhibit dissociable interindividual variability and influence perception of different visual features separately. An alternative hypothesis is that the GABA levels of different cortical regions may covary as a result of common embryonic origins or shared subcortical GABAergic projections (Dammerman et al., 2000; Jinno et al., 2007; Picardo et al., 2011; Caputi et al., 2013; Chen and Kriegstein, 2015) and may influence perception of different visual features concurrently.

To test these two alternative hypotheses, we studied how the GABA levels of parietal and occipital cortices related to interindividual differences in size, orientation, and brightness perception. Occipital cortex contains a map of orientation preference in which individual neurons respond preferentially to specific orientation and neighboring neurons to adjacent orientations; by contrast, parietal cortex contains a map of size preference in which individual neuronal populations respond preferentially to specific size of a visually presented object and neighboring neurons to adjacent sizes (Yacoub et al., 2008; Harvey et al., 2015). Since neurotransmitters are contained and released at synapses, the GABA level of a cortical region may influence visual perception through synaptic connections within the region, which link neighboring neurons of similar feature preferences. These intraregional lateral connections underlie contextual illusions where the perceived feature (e.g., orientation, size) of a visual stimulus is modulated by the stimulus surrounding it (Cannon and Fullenkamp, 1996; Kapadia et al., 2000; Stettler et al., 2002; Bosten and Mollon, 2010; Song et al., 2013a). We therefore used contextual illusion as a perceptual assay, hypothesizing that selective correlation may be observed between the GABA level of a cortical region and the contextual illusion for visual features topographically mapped in this cortical region. Specifically, parietal and occipital GABA levels may correlate selectively with the magnitude of size and orientation illusion.

## Materials and Methods

### 

#### 

##### Participants.

Thirty-seven healthy volunteers (age range, 20–40 years; all males; females were ineligible due to the menstrual cycle) gave written informed consent to participate in this study that was approved by the local ethics committee, De Videnskabsetiske Komitéer for Region Midtjylland, Denmark. All participants had normal or corrected-to-normal vision and no neurological or psychiatric history. The MRS data of four participants were contaminated by the signal from lipids, and the psychophysics data of three participants were outliers of the normal distribution (Shapiro-Wilk test). These data were therefore excluded from further analysis.

##### Magnetic resonance spectroscopy (MRS) measure of GABA.

Neuroimaging experiments took place in a Siemens Trio 3T MRI scanner. Structural MRI data were acquired using a T1-weighted MPRAGE sequence (TR, 2420 ms; TE, 3.7 ms; resolution, 1 mm isotropic; scanning time, 5.5 min) and were used to guide the voxel placement in MRS. Resting GABA measures were acquired respectively from a 2 cm isotropic voxel in the parietal lobe (TR, 2500 ms; TE, 68 ms; 240 edit on and edit off averages; scan time, 20 min) and a 3 cm isotropic voxel in the occipital lobe (TR, 2500 ms; TE, 68 ms; 96 edit on and edit off averages; scan time, 8 min) using MEGA-PRESS method (Mescher et al., 1998; Edden and Barker, 2007). To compensate for the size differences between the two voxels, the parietal voxel had a longer scan time (20 min) than the occipital voxel (8 min). An even longer scan time (40 min) could lead to a better compensation; however, the subject motion would be a drawback. The MRS measure of resting GABA varies little across days or even months (Evans et al., 2010; Near et al., 2014). The high test–retest reliability suggests that the scanning order will not bias the measures. Nevertheless, to minimize the between-subject variance of no interest, we kept the scanning order identical for all participants, collecting data for the occipital voxel first and the parietal voxel second. The parietal voxel was placed on the anterior part of the superior parietal lobe with its anterior border in parallel with the postcentral gyrus. The occipital voxel was placed to cover the calcarine sulcus bilaterally with its anterior border in alignment with the parietal-occipital sulcus. Care was taken to avoid the inclusion of the scalp and/or the tentorium cerebelli in the voxels.

The MEGA-PRESS method measures the level of GABA through the acquisition of the following two spectra: one with an editing pulse targeting the C3-GABA peak at 1.9 ppm (edit on); and one with an editing pulse targeting the water peak on the symmetrical side at 7.5 ppm (edit off). By averaging the two spectra, the creatine (Cr) peak at 3.0 ppm was quantified. By subtracting the two spectra, the C4-GABA peak at 3 ppm was quantified. This C4-GABA peak is often referred to as GABA^+^, since a coupled macromolecule (MM) resonance at 3 ppm is coedited and contributes to the measured signal. Due to the limitation of the MEGA-PRESS sequence, the exact MM contribution is difficult to estimate or remove. A theoretical model has been proposed to subtract MM contribution *post hoc* (Murdoch and Dydak, 2011). Nevertheless, this technique could introduce additional variability into the estimated GABA values and is thus rarely used (Mullins et al., 2014, their discussion). Newer sequences such as MEGA-SPECIAL (Near et al., 2011) and SPECIAL (Near et al., 2013) aim to remove MM contribution by editing and modeling, respectively. However, both sequences have other drawbacks such as the imperfect lipid suppression. The raw GABA value is subject to bias from day-to-day scanner-related variation. For an unbiased estimate of GABA, a normalization of raw GABA value to Cr is typically applied (Mullins et al., 2014), since Cr resonates around the same frequency (3 ppm) as GABA and is not affected by disturbances that depend on the resonance frequency. The ratio GABA^+^/Cr was calculated to quantify GABA level.

**Figure 1.**
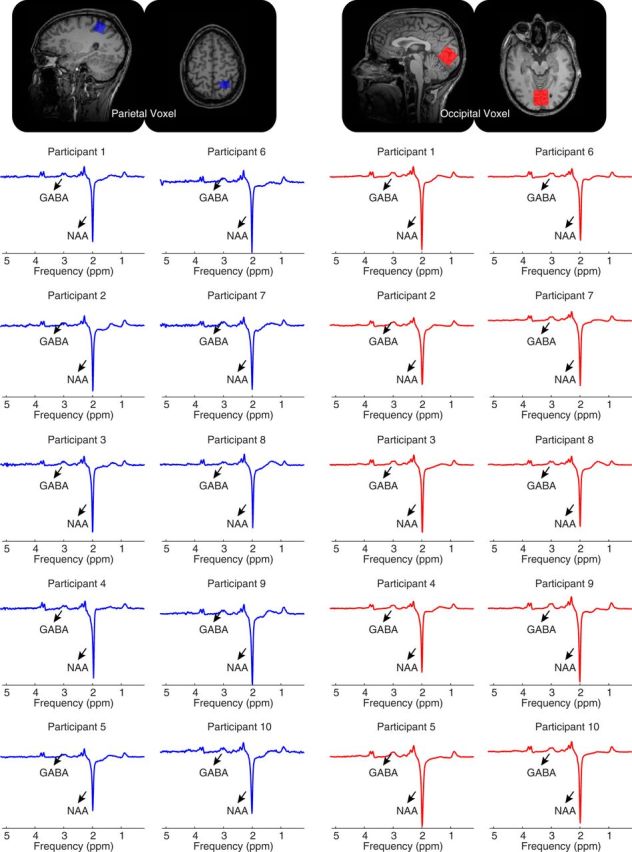
Magnetic resonance spectroscopy (MRS). In two separate experiments, MRS measure of GABA level was acquired respectively, from a parietal voxel (blue) placed on the anterior part of the superior parietal lobe with its anterior border parallel to the postcentral gyrus, and a occipital voxel (red) placed to cover the calcarine sulcus bilaterally with its anterior border in alignment with the parietal-occipital sulcus. Examples of MRS spectra from 10 randomly selected participants are shown. The GABA peak is seen at 3 ppm and the inverted NAA peak at ∼2 ppm.

The analysis of MRS data was performed by author J.U.B., who was blind to the psychophysics data, and the analysis constituted part of a database that have been reported in previous studies (Near et al., 2014; Sandberg et al., 2014, 2016;). The MRS data were first preprocessed in MATLAB with FID-A software for motion corruption removal, drift correction, and phasing, and then were analyzed in jMRUI software with the AMARES package (Mescher et al., 1998; Edden and Barker, 2007; Simpson et al., 2017). Data were visually inspected for noise, line broadening, voxel misplacement, and lipid contamination. Four participants who had spectra with large lipid contamination failed to pass the visual inspection and were excluded from further analysis. The quality of the included spectra was evaluated by calculating signal-to-noise ratio (SNR), line width, and fit uncertainty. Examples of typical spectra are shown in Figure 1. SNR was calculated using the difference spectrum following the phase adjustment such that the *N*-acetylaspartate (NAA) peak was upright with a phase of 0°. The signal was calculated as the maximal intensity of the NAA peak in the difference spectrum; noise was calculated as the SD of the noise in the signal-free spectrum following a baseline correction to remove any first- and second-order baseline variations. The SNR was 108 for the parietal voxel and 226 for the occipital voxel. Line width was calculated by measuring the full-width at half-maximum of the NAA peak in the difference spectrum. The mean line width was 4.8 Hz for the parietal voxel and 5.4 Hz for the occipital voxel. Fit uncertainty was measured as the SD/amplitude ratio output by jMRUI. The mean SD/amplitude ratio was 0.04 for the parietal voxel and 0.03 for the occipital voxel.

##### Psychophysics measure of contextual illusion.

Psychophysics experiments took place in a dark room. Visual stimuli were presented on a 17 inch LCD monitor (spatial resolution, 1024 × 768 pixels; temporal resolution, 60 Hz) and viewed through a chin rest. The magnitudes of size illusion (Ebbinghaus illusion), orientation illusion (tilt illusion), and brightness illusion (simultaneous contrast illusion) were measured in separate experiments. The size illusion stimulus comprised two white circles (1° diameter), a reference circle surrounded by 16 small white circles (0.2° diameter) and a test circle by seven large white circles (2° diameter), that were presented simultaneously for 500 ms on two sides of the fixation (3.85° eccentricity) with randomized spatial order. The orientation illusion stimulus comprised two circular gratings (45° orientation, 1.5° diameter, 2.5 cycles/° spatial frequency, 100% contrast), a reference grating surrounded by an annular grating (60° orientation, 4.5° diameter, 2.5 cycles/° spatial frequency, 100% contrast) and a test grating with no surround. The brightness illusion stimulus comprised two gray circles (50% luminance, 1.5° diameter), a reference circle surrounded by a white annulus (4.5° diameter) and a test circle by a black annulus (4.5° diameter).

To minimize the confounding factors such as decision factors (Vogels and Orban, 1986; Gold and Ding, 2012), we kept the psychophysical procedures identical for all three illusions. Participants first performed a match-to-standard session in which they manually adjusted the size, orientation, or luminance of the test stimulus until it matched the perceived size, orientation, or luminance of the reference stimulus, and a visual discrimination session in which the size, orientation, and luminance discrimination threshold was measured through standard 2-up-1-down staircase procedures. The point of subjective equality measured from the match-to-standard session and the visual discrimination threshold measured from the staircase session were used to guide the choices of stimulus parameters in the subsequent two-alternative forced-choice session. There, participants were asked on 112 trials to judge which central stimulus was larger (for size illusion), more tilted (for orientation illusion), or brighter (for brightness illusion). The size, orientation, or luminance of the reference stimulus was kept constant; that of the test stimulus was varied among seven values (16 trials per value) around the point of subjective equality acquired from the match-to-standard session, with a step size equal to the visual discrimination threshold.

The data from the two-alternative forced-choice session were fitted with psychometric function to measure the 50% threshold point where the two central stimuli appeared perceptually equal despite their physical difference. The goodness of fit (*R*^2^) was 0.963 ± 0.033 for orientation illusion, 0.956 ± 0.041 for size illusion, and 0.960 ± 0.033 for brightness illusion. The goodness of fit did not differ significantly between illusions (size illusion vs orientation illusion: *t*_(29)_ = 1.03, *p* = 0.313; size illusion vs brightness illusion: *t*_(29)_ = 0.47, *p* = 0.640; orientation illusion vs brightness illusion: *t*_(29)_ = 0.28, *p* = 0.785) or correlate significantly with GABA (size illusion and parietal GABA: *r* = −0.194, *p* = 0.304; size illusion and occipital GABA: *r* = 0.143, *p* = 0.451; orientation illusion and parietal GABA: *r* = 0.244, *p* = 0.194; orientation illusion and occipital GABA: *r* = 0.142, *p* = 0.456; brightness illusion and parietal GABA: *r* = −0.224, *p* = 0.234; brightness illusion and occipital GABA: *r* = 0.174, *p* = 0.359). The physical difference between the two central stimuli at the 50% threshold point was calculated to quantify the illusion magnitude.

To account for the influence of Weber's law (Shen, 2003), we used the log transform of the illusion magnitude and the semi-log plots (see Figs. 3, 4, 5) to assess interindividual differences. Since the magnitude of orientation illusion is subject to the oblique effect (Clifford, 2014), we performed additional control experiments in a group of 20 healthy volunteers (age range, 21–35 years; 11 females) to test the influence of stimulus orientation (cardinal vs oblique) on the measure of interindividual differences. We found that although the illusion magnitude was weaker for the cardinal condition than the oblique condition (*t*_(19)_ = 20.362, *p* < 10^−13^), the illusion magnitude was highly correlated between the two conditions (*r* = 0.866, *p* < 10^−6^). This observation suggested that interindividual differences in orientation illusion magnitude were not biased by the oblique effect.

##### Statistics.

Pearson's correlation can capture the linearity in the relation between two variables, whereas Spearman's rank correlation can only reflect whether two variables are monotonically related or not. For example, Spearman's correlation coefficient will return the same result of 1 for two variables that both monotonically increase, regardless of whether their rates of increase are linearly or nonlinearly correlated; by contrast, Pearson's correlation coefficient can capture the difference between these two conditions. As such, Pearson's correlation coefficient is a more suitable test for comparing the correlation coefficient between different experimental conditions. Application of Pearson's correlation requires the data to satisfy normal distribution. The Shapiro–Wilk test failed to refute the assumption of normality for parietal GABA level [coefficient of concordance (W) = 0.952, *p* = 0.187], occipital GABA level (W = 0.962, *p* = 0.295), size illusion magnitude (W = 0.937, *p* = 0.072), orientation illusion magnitude (W = 0.985, *p* = 0.942), or brightness illusion magnitude (W = 0.960, *p* = 0.314). Therefore, Pearson's correlation was used throughout this study to test the relations between variables, with age regressed out.

## Results

**Figure 2.**
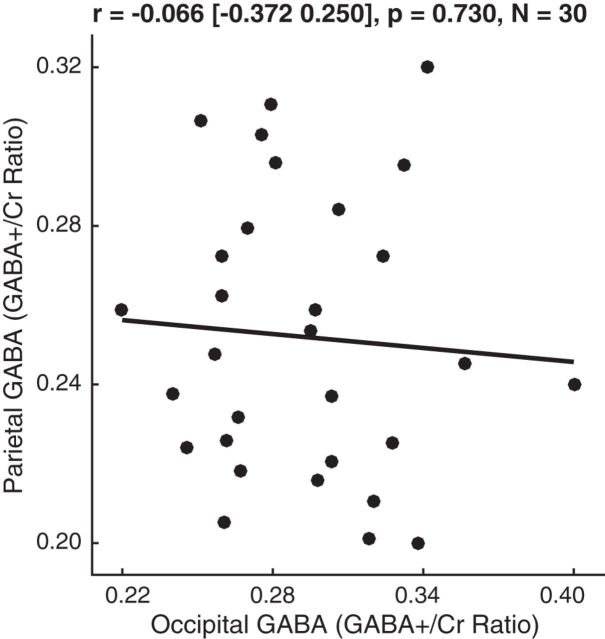
Parietal and occipital GABA. The GABA levels of parietal and occipital cortices were plotted against each other, illustrating a lack of interindividual correlation between the two variables. Each data point represents a participant. Statistical values reflect Pearson's correlation coefficient and 95% bootstrap confidence interval.

We found that the GABA level in parietal cortex (0.252 ± 0.035) and the GABA level in occipital cortex (0.299 ± 0.042) exhibited dissociated interindividual variability [Fig. 2; *r* = −0.066 (95% CI, −0.372 to 0.250); *p* = 0.730, *N* = 30]. Subsequently, we studied how parietal GABA level versus occipital GABA level contributed to interindividual differences in size illusion (Ebbinghaus illusion), orientation illusion (tilt illusion), and brightness illusion (simultaneous contrast illusion).

**Figure 3.**
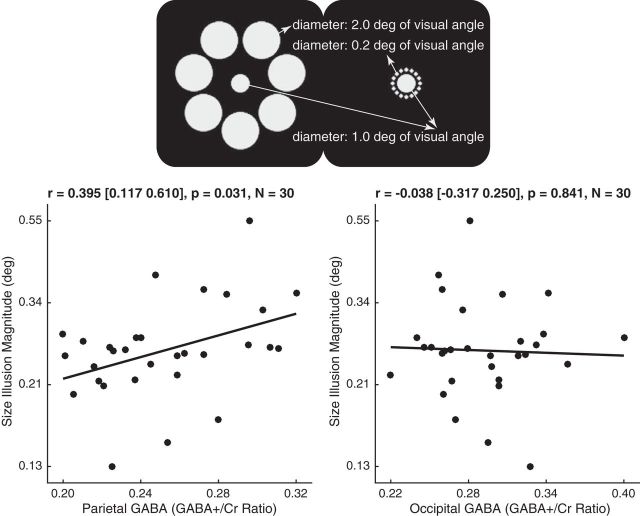
GABA level and size illusion. In the Ebbinghaus illusion, two physically identical central circles appear to have different perceived sizes as a result of the surrounding context of either smaller or larger circles. The magnitude of the Ebbinghaus illusion for each participant was plotted in semi-log graph against their parietal or occipital GABA level, illustrating a positive correlation between size illusion magnitude and parietal GABA level, as well as a lack of significant correlation between size illusion magnitude and occipital GABA level. Each data point represents a participant. Statistical values reflect Pearson's correlation coefficient and 95% bootstrap confidence interval.

Across individuals, we observed a positive correlation between the magnitude of size illusion and the level of parietal GABA [Fig. 3; *r* = 0.395 (95% CI, 0.117–0.610); *p* = 0.031, *N* = 30]. By contrast, we did not observe any significant correlation between the magnitude of size illusion and the level of occipital GABA [Fig. 3; *r* = −0.038 (95% CI, −0.317 to 0.250); *p* = 0.841, *N* = 30]. Moreover, compared with occipital GABA level, parietal GABA level showed a significantly higher correlation with size illusion magnitude (*t*_(27)_ = 2.369, *p* = 0.018). These results suggest a selective correlation between size illusion and parietal GABA.

**Figure 4.**
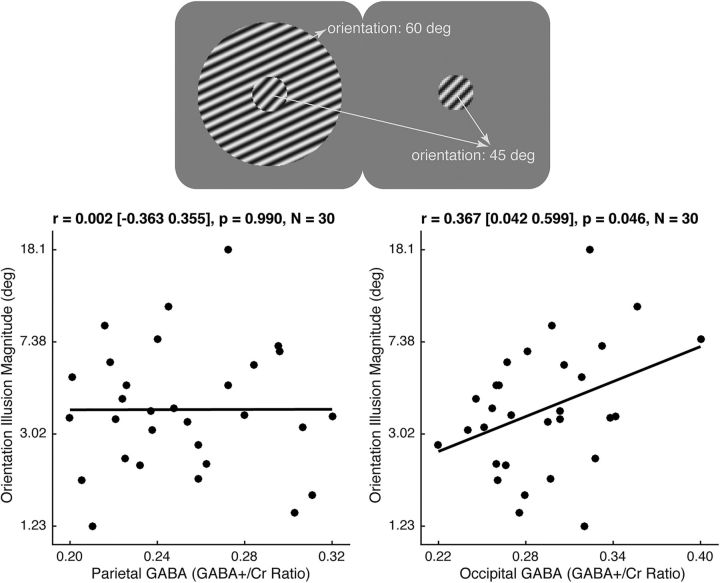
GABA and orientation illusion. In the tilt illusion, two physically identical central gratings appear to have different perceived orientations as a result of their immediate surroundings. The magnitude of the tilt illusion for each participant was plotted in semi-log graph against their parietal or occipital GABA level, illustrating a positive correlation between orientation illusion magnitude and occipital GABA level, as well as a lack of significant correlation between orientation illusion magnitude and parietal GABA level. Each data point represents a participant. Statistical values reflect Pearson's correlation coefficient and 95% bootstrap confidence interval.

Conversely, across individuals, the magnitude of orientation illusion exhibited a positive correlation with the level of occipital GABA [Fig. 4; *r* = 0.367 (95% CI, 0.042–0.599); *p* = 0.046, *N* = 30], but not with the level of parietal GABA [Fig. 4; *r* = 0.002 (95% CI, −0.363 to 0.355); *p* = 0.990, *N* = 30]. Moreover, occipital GABA level had a significantly greater correlation with orientation illusion magnitude than did parietal GABA level (*t*_(27)_ = 1.990, *p* = 0.047). These results suggest a selective correlation between orientation illusion and occipital GABA.

**Figure 5.**
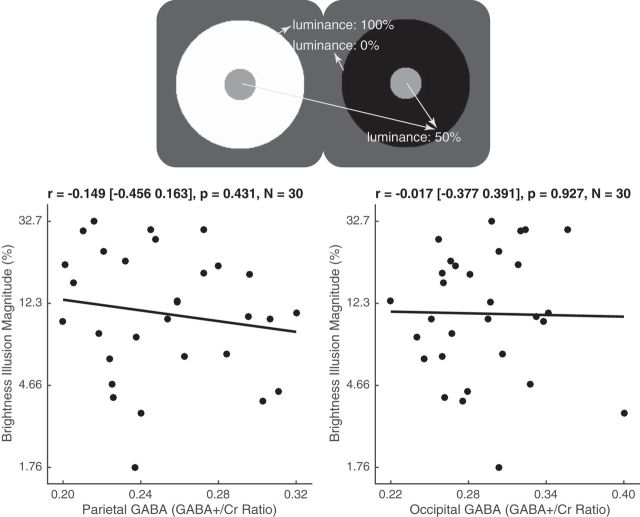
GABA level and brightness illusion. In the simultaneous contrast illusion, two physically identical central circles appear to have different brightness as a result of their immediate surroundings. The magnitude of simultaneous contrast illusion for each participant was plotted in semi-log graph against their parietal or occipital GABA level, illustrating a lack of significant correlation between brightness illusion magnitude and either parietal or occipital GABA level. Each data point represents a participant. Statistical values reflect Pearson's correlation coefficient and 95% bootstrap confidence interval.

For the brightness illusion, we did not observe any significant correlation across individuals between the illusion magnitude and parietal GABA level [Fig. 5; *r* = −0.149 (95% CI, −0.456 to 0.163); *p* = 0.431, *N* = 30] or occipital GABA level [Fig. 5; *r* = −0.017 (95% CI, −0.377 to 0.391); *p* = 0.927, *N* = 30]. Accordingly, the correlation between parietal GABA level and brightness illusion magnitude was not significantly different from the correlation between occipital GABA level and brightness illusion magnitude (*t*_(27)_ = 0.690, *p* = 0.490).

## Discussion

Together, our study reveals a region- and feature-dependent influence of neurotransmitter level on human visual perception. We show that interindividual variability in parietal GABA level correlated with size illusion magnitude but not with orientation or brightness illusion magnitude; in contrast, interindividual variability in occipital GABA level correlated with orientation illusion magnitude but not with size or brightness illusion magnitude. Our findings suggest that interindividual variability in GABA does not reflect a general variability in visual inhibition; instead, the GABA levels of different cortical regions have selective influence on perception of different visual features. This influence is likely to be exerted through lateral connections within the cortical region and is observed specifically for visual features mediated by such connections.

In occipital cortex, neurons exhibit orientation preference such that their response is the strongest for a preferred orientation and gradually decays as the stimulus orientation deviates from this preferred orientation (Ringach et al., 2002). Neurons preferring adjacent orientations are cortically adjacent to one another and are connected by intraregional lateral connections (Yacoub et al., 2008; Bock et al., 2011; Li et al., 2012). This topographical organization of lateral connections allows the orientation preference of neurons to be modulated by the activity of their adjacent neurons and the level of occipital GABA to affect the degree of modulation (Burr et al., 1981; Morrone et al., 1987; Eysel et al., 1990; Gilbert et al., 1996; Fitzpatrick, 2000; Stettler et al., 2002; Smith et al., 2006; Chavane et al., 2011). This neural-level modulation may then give rise to perceptual-level modulation, where the perceived orientation of a stimulus is modulated by the orientation of the stimulus surrounding it (Schwartz et al., 2007; Song et al., 2013a). If so, the correlation between the magnitude of orientation illusion and the level of occipital GABA could be a perceptual reflection of the GABA influence on neural orientation preference.

Whereas orientation preference is topographically mapped in occipital cortex with neurons preferring more similar orientations being more highly connected, there is no topographic map of size preference in occipital cortex (Swindale, 2000; Chklovskii and Koulakov, 2004). As such, a local GABA influence, exerted through lateral connections within occipital cortex, is likely to be specific to orientation illusion and not generalizable to size illusion. Just as the topographic map of orientation preference is prominent in occipital cortex (Wolf and Geisel, 1998; Yacoub et al., 2008; Kaschube et al., 2010), a topographic map of size preference exists in parietal cortex where individual neuronal populations respond preferentially to a specific size and adjacent neurons respond to adjacent sizes (Harvey et al., 2015). By contrast, there is no map of orientation preference in parietal cortex. Therefore, a local GABA influence, exerted through lateral connections within parietal cortex, would be specific to size illusion and not generalizable to orientation illusion. Similar to the orientation preference and size preference in cortical neurons, neurons in the retina exhibit preference for stimulus luminance and are topographically connected by their luminance preference. Possibly, the interindividual differences in brightness illusions may associate with interindividual variability in retinal GABA (Lukasiewicz and Shields, 1998; Wu, 2010). Moreover, since neural responses to visual features are modulated not only by intraregional lateral connections but also by inter-regional feedback connections (Fitzpatrick, 2000; Smith et al., 2006), the lack of correlation between brightness illusion and occipital or parietal GABA level could also indicate a predominant contribution of inter-regional (as opposed to intraregional) factors to this illusion (Kinoshita and Komatsu, 2001; Perna et al., 2005).

This proposal, that the GABA level of a cortical region influences perception of visual features topographically mapped in this region, would be able to explain the reported correlations between occipital GABA level and orientation discrimination threshold (Edden et al., 2009). The intraregional modulation exerted through lateral connections may not only shift neural orientation preference and give rise perceptual shifts in orientation illusion, but also sharpen neural orientation preference (orientation tuning) and give rise to perceptual sharpening in orientation discrimination (Ben-Yishai et al., 1995; Somers et al., 1995; Orban and Vogels, 1998; Song et al., 2013b; Song et al., 2015). As such, the influence of occipital GABA level on orientation illusion could be mirrored in orientation discrimination (Song et al., 2013b). In addition to orientation preference, ocular preference is also topographically mapped in occipital cortex, where individual neurons respond preferentially to stimulus from a specific eye and adjacent neurons to opposite eyes (Menon et al., 1997; Dechent and Frahm, 2000; Adams et al., 2007). There, lateral connections would link neurons with opposite ocular preference, allowing the GABA influence on orientation perception to generalize to binocular perception. This would explain the reported decrease in both occipital GABA level and interocular suppression after monocular deprivation (Lunghi et al., 2015).

Our proposal, that the GABA level of a cortical region influences perception of visual features topographically mapped in this region, further predicts a correlation between parietal GABA and numerosity perception. Just as occipital cortex is crucial for processing low-level visual features and contains maps of orientation preference and ocular preference, parietal cortex is important for processing high-level visual features and contains maps of size preference and numerosity preference (Walsh, 2003; Chklovskii and Koulakov, 2004; Pinel et al., 2004; Dehaene and Cohen, 2007; Roitman et al., 2007; Dormal et al., 2008; Bueti and Walsh, 2009; Cohen Kadosh and Walsh, 2009; Nieder and Dehaene, 2009; Roitman et al., 2012; Harvey et al., 2013; Harvey et al., 2015). The lateral connections in parietal cortex are likely to link neighboring neurons with similar numerosity preference, which would allow parietal GABA to influence numerosity discrimination and numerosity illusion (Pinel et al., 2004; Almeida et al., 2007; Dormal et al., 2008; Bosten and Mollon, 2010). While the posterior part of the cortex (e.g., occipital cortex, parietal cortex) is involved in sensory processing, a topographic map of conceptual knowledge was discovered in prefrontal cortex, suggesting a potential role of frontal GABA in conceptual categorization (Constantinescu et al., 2016). It would thus be of interest for future studies to test the links between parietal GABA and numerosity perception, or between frontal GABA and conceptual categorization.
